# Special Issue “Small Molecules, Influence of Molecular Pathways 2.0”

**DOI:** 10.3390/ijms24119508

**Published:** 2023-05-30

**Authors:** Eng Shi Ong

**Affiliations:** Department of Science, Singapore University of Technology and Design, Singapore 487372, Singapore; engshi_ong@sutd.edu.sg

Small molecules play an important role in extracting energy from cells, synthesising new macromolecules, and indicating metabolic shift and other processes (Figure 1) [1]. It was noted that more than 30 chemical entities were approved by the U.S. Food and Drug Administration during 2021 [2]. In addition, many new small chemical entities have been observed to have notable structures and novel clinical applications [2,3,4]. Small molecules have been found to show promising leads in the development of new drugs for the improvement of human health [1,2]. On top of that, small chemical entities, such as polyphenols, carotenoids and others, found in medicinal plants and functional foods have shown a range of biological effects, such as anti-inflammatory, anti-aging and other properties [5,6].

One such small molecule is trimethylamine N-oxide (TMAO), which is a biologically active gut microbiome-derived dietary metabolite. A high circulating plasma TMAO level has been proposed to correlate with diseases, such as atherosclerosis and hypertension, and metabolic disorders, such as diabetes. At the same time, TMAO can induce endothelial dysfunction in cardio-metabolic diseases. The inflammation and oxidative stress caused by TMAO is mainly driven by the activation of foam cells, the upregulation of cytokines and other inflammatory mediators, and an increased production of reactive oxygen species (ROS) [6].

The metabolic shift seen in Figure 1 can often be characterized by small molecules, such as glucose, amino acids, fatty acids and others. The administration of small chemical entities, such as TMAO and others, results in metabolic shift in biofluids and tissue samples. Small molecules as such can be used as biomarkers to determine the effects of drugs and functional foods [5,7]. In short, the clinical effects of drugs and small molecules in functional foods have been proposed to correlate with the metabolic shift of small molecules in human biofluids and tissue samples [5,7].

The current Special Issue provides an in-depth understanding of how small molecules interact with biological systems and their effects on metabolites. This Special Issue also showcases research on how small molecules present in biofluids and tissues in animal and human models can be applied for the improvement of human health.

## Figures and Tables

**Figure 1:**
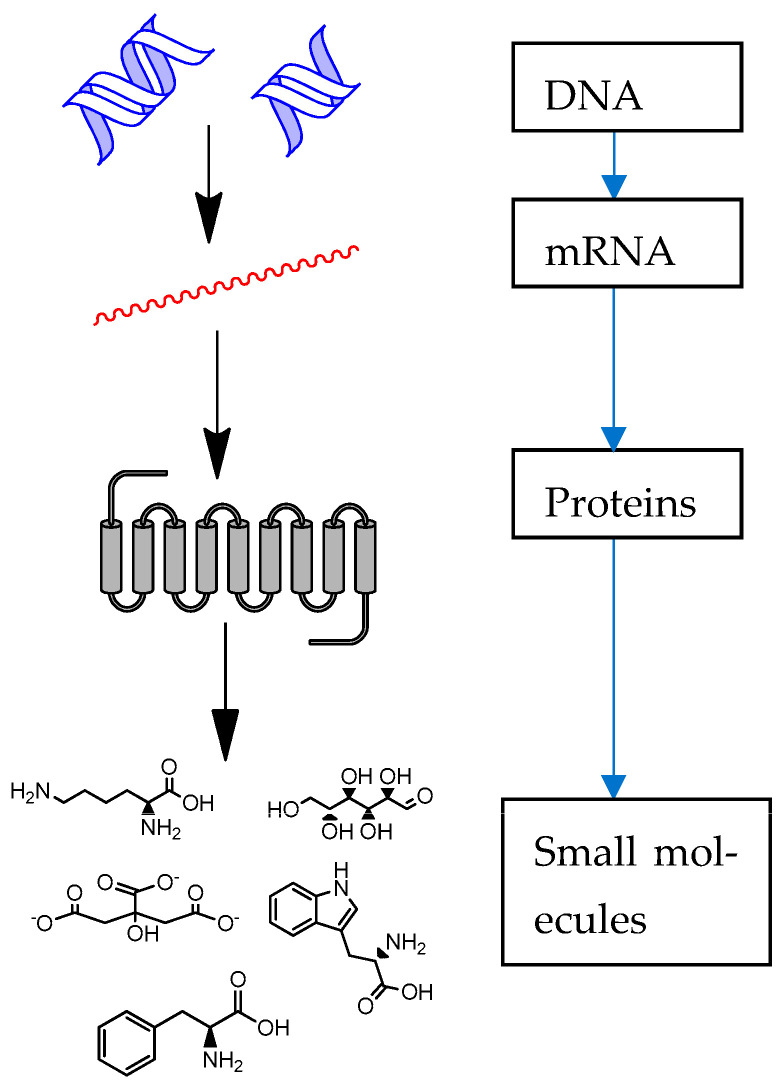
Roles of small molecules in extracting energy from cells, synthesising new macromolecules, and indicating metabolic shift and other processes.

## References

[1] Leo C.H., Ou J.L.M., Ong E.S., Qin C.X., Ritchie R.H., Parry L.J., Ng H.H. (2023). Relaxin elicits renoprotective actions accompanied by increasing bile acid levels in streptozotocin-induced diabetic mice. Biomed. Pharmacother..

[2] Yuan S., Wang D.S., Liu H., Zhang S.N., Yang W.G., Lv M., Zhou Y.X., Zhang S.Y., Song J., Liu H.M. (2023). New drug approvals for 2021: Synthesis and clinical applications. Eur. J. Med. Chem..

[3] Mullard J.A. (2022). 2021 FDA approvals, Nat. Rev. Drug Discov..

[4] (2022). Advancing Health through Innovation: New Drug Therapy Approvals 2021.

[5] Xu D.P., Li Y., Meng X., Zhou T., Zhou Y., Zheng J., Zhang J.J., Li H.B. (2017). Natural Antioxidants in Foods and Medicinal Plants: Extraction, Assessment and Resources. Int. J. Mol. Sci..

[6] Shanmugham M., Bellanger S., Leo C.H. (2023). Gut-Derived Metabolite, Trimethylamine-N-oxide (TMAO) in Cardio-Metabolic Diseases: Detection, Mechanism, and Potential Therapeutics. Pharmaceuticals.

[7] Yuliana N.D., Hunaefi D., Goto M., Ishikawa Y.T., Verpoorte R. (2021). Measuring the health effects of food by metabolomics. Crit. Rev. Food Sci. Nutr..

